# Acquired thrombotic thrombocytopenic purpura in a patient with *plasmodium vivax* malaria: A case report

**DOI:** 10.1590/0037-8682-0014-2024

**Published:** 2024-09-02

**Authors:** Daren Esteban Araque Gualtero, Diego Augusto Moreno Diaz, Julie Melissa Mogollón, Andrés Felipe Gómez Rueda

**Affiliations:** 1 Facultad de Medicina de la Universidad Industrial de Santander, Bucaramanga, Santander, Colombia.

**Keywords:** Thrombotic thrombocytopenic purpura, Microangiopathic hemolytic anemia, *Plasmodium vivax* malaria

## Abstract

Acquired thrombotic thrombocytopenic purpura (TTP) is a rare life-threatening disorder characterized by microangiopathic hemolytic anemia, severe thrombocytopenia, and organ damage. We present the case of a 71-year-old man initially diagnosed with malaria-like symptoms and displaying markers of microangiopathic hemolytic anemia, severe thrombocytopenia, renal injury, and neurological impairment. Despite antimalarial treatment, acquired TTP was suspected. Plasma exchange and immunosuppressive therapy led to clinical improvement, normalizing the platelet count and hemolytic profile. Diagnostic confirmation revealed significantly reduced ADAMTS13 levels. Following the proposed treatment, the patient's ADAMTS13 levels normalized. This case illustrates acquired TTP linked to uncomplicated *Plasmodium vivax* malaria.

## INTRODUCTION

Thrombotic thrombocytopenic purpura (TTP) is an extremely rare disease with a high mortality rate and tendency to recur. Even with early and appropriate treatment, up to 20% of patients die, and half experience relapse throughout their lives^1^. In Colombia, the disease is under-reported; therefore, its prevalence has not been clearly defined. Studies are mostly limited to reports or case series. Recently, a series with the largest population of TTP in Colombia, with 19 patients, was published. It describes the main clinical features and treatment approaches in our environment, where the main clinical manifestations were neurological symptoms (73.6%), renal involvement (68.4%), gastrointestinal involvement (52.6%), and fever (47.3%), associated with systemic lupus erythematosus in 47.6% and idiopathic in 31.5% of patients^2^.

TTP is characterized by an abnormal accumulation of unusually large multimers of von Willebrand factor (VWF) caused by insufficient or absent activity of a disintegrin and metalloprotease with a thrombospondin type 1 motif, member 13 (ADAMTS13), which has the function of fragmenting VWF multimers acquired by autoantibodies (TTPa) or inherited due to mutations in the *ADAMTS13* gene in the congenital form. This produces a generalized deposition of platelet-rich microthrombi in the microvasculature, which is known as thrombotic microangiopathy and is clinically characterized by microangiopathic hemolytic anemia, in which schistocytes or fragments of red blood cells are recognized in the peripheral blood, associated with thrombocytopenia due to consumption and tissue damage with organ dysfunction. TTPa often follows an acute episode of inflammation and/or infection that may trigger the formation of autoantibodies due to the loss of immune tolerance^3^. Diagnosis was confirmed by measuring ADAMTS13 levels, which must be less than 10%. The demonstration of inhibitors against the enzyme is not available in most centers, and it is suggested to be performed mainly in cases where there are substantial doubts about the etiology. Currently, the treatment of the disease is based on the early removal of antibodies and replacement of the ADAMTS13 enzyme by plasma exchange; the blockade of platelet adhesion with caplacizumab, a nano-antibody recently available in Colombia that aims to prevent ischemic injury with consequent multiorgan dysfunction; and immunosuppression obtained with high doses of steroids and rituximab to achieve remission and reduce the risk of relapse^4^.

However, reduced ADAMTS13 levels are not limited to TTPa; severely decreased ADAMTS13 activity has been demonstrated in other thrombocytopenic diseases, such as sepsis, disseminated intravascular coagulation, and complicated malaria infection. However, the enzyme values were typically greater than 10% for these entities. This explains the similarities between the microvascular disease in malaria and TTPa^5^ ^,^ ^6^.

A thorough investigation was performed across multiple databases, including MEDLINE via PubMed, Cochrane Library, LILACS, SciELO, and PubMed Central, with no temporal restrictions. The search was conducted in October 2023 using a combination of specific descriptors (highlighted in bold), synonyms, natural language, and Boolean operators with the following query structure: (thrombotic thrombocytopenic purpura) AND (*Plasmodium vivax* malaria). Within the existing literature, the coexistence of thrombotic thrombocytopenic purpura alongside malaria caused by *P. vivax* has not been documented. In contrast, such coexistence has been reported in *Plasmodium falciparum*. This study presents an initial report on the simultaneous presence of these two entities. Consequently, it is essential to investigate the potential precipitating or epiphenomenal factors to elucidate the underlying reasons for the association between both diseases. We present the case of a patient with a confirmed diagnosis of malaria caused by *P. vivax*, in whom, in the absence of improvement with the specific treatment established, differential diagnoses were evaluated with subsequent confirmation of TTPa. 

## CASE REPORT

A 71-year-old male patient from Sur de Bolívar, Colombia, with no known medical history, was admitted to the hospital from a lower level of care with a one-day history of psychomotor agitation and incoherent speech. Extra-institutional laboratory tests showed moderate anemia of normal volume and severe thrombocytopenia with a thick positive smear for *P. vivax*. Due to the neurological involvement, they suspected complicated malaria and referred the patient to a higher level of care for management in the intensive care unit (ICU).

On admission to our institution, the patient was neurologically disoriented and somnolent without signs of meningeal irritation or focalization. The remaining physical examinations did not reveal any alterations. A simple brain scan revealed no pathological findings. Laboratory tests revealed elevated blood urea nitrogen, severe anemia with markers of non-autoimmune hemolysis, and schistocytes in the peripheral blood associated with severe thrombocytopenia. The thick control smear was negative. (Table 1). Complicated malarial syndrome was considered to be due to cerebral and hematological involvement, the latter suggestive of thrombotic microangiopathy secondary to the infectious process. The patient was admitted to the ICU, and treatment with intravenous artemisinin derivatives was initiated as first-line management for complicated malaria according to the Colombian guidelines, as well as supportive transfusion management.

**TABLE 1:** Laboratory results on admission.

Parameters	Result
Hemoglobin (g/dL)	5.8
Hematocrit (%)	18.4
Mean Corpuscular Volume (fL)	104
White blood cells (x10^3^/µL)	6.98
Neutrophil (%)	57.8
Lymphocytes (%)	32.2
Platelets (x10^3^/µL)	9
Total bilirubin (mg/dL)	1.63
Indirect bilirubin (mg/dL)	1.38
Thick drop	Negative
VDLR	No reactive
Prothrombin time (s)	17.7
INR	1.29
Partial thromboplastin time (s)	29.8
Creatinine (mg/dL)	1.29
Urea Nitrogen (mg/dL)	36.7
Alkaline phosphatase (U/L)	46
AST-ALT (mg/dL)	130 - 37
Folic Acid (ng/mL)	10.8
Vitamin B12 (pg/mL)	625
Peripheral blood smear	PolychromatophiliaSchistocytes 2+

Despite completing treatment with artesunate, the patient continued to have neurological, renal, and hematological abnormalities, with high transfusion requirements (Table 2). Therefore, given the persistence of laboratory findings with a microangiopathic hemolytic anemia profile (Table 2, day-3) and a PLASMIC score with a high probability of severe ADAMTS13 deficiency, probable acquired thrombotic thrombocytopenic purpura was considered, starting management with plasma exchange and high-dose steroids prior to recording ADAMTS13 levels.

**TABLE 2:** Paraclinical tests evolution during hospitalization.

Parameters	Day 3	Day 7	Day 15
Hemoglobin (g/dL)	6.5	7.8	8.5
Hematocrit (%)	21.6	26.2	27
Mean Corpuscular Volume (fL)	100.9	106.9	105
White blood cells (x10^3^/µL)	8.27	7.96	3.12
Neutrophil (%)	51.7	89.6	78.5
Lymphocytes (%)	20.6	7.4	14.1
Platelets (x10^3^/µL)	17	59	174
Total bilirubin (mg/dL)	7.60	2.60	0.26
Indirect bilirubin (mg/dL)	4.39	1.65	0.19
Lactate dehydrogenase (U/L)	2853	518	191
Direct Coombs test	Negative		
Thick drop	Negative		
Reticulocyte count (%)	19.25	18.11	3.36
Complement C4 (mg/dL)	24.80		
Creatinine (mg/dL)	1.52	1.74	1.24
Urea Nitrogen (mg/dL)	54.2	64.20	27.3
Vitamin B12 (pg/mL)	625		
Peripheral blood smear	Schistocytes 3+	Schistocytes 3+	Schistocytes 1+

After the initiation of therapeutic apheresis therapy and high-dose steroids, the patient showed substantial improvement in neurological symptoms, renal function, and hemolysis profile (Table 2, Day 7). The diagnosis of TTP was confirmed with a report of <0.1% ADAMTS13. Therefore, immunosuppression for relapse prevention was associated with rituximab. The patient received a total of (18 plasma exchanges and three doses of rituximab) (Table 2, day 15), achieving normalization of the platelet count, improvement and stability of hemoglobin, normalization of the hemolysis profile and renal injury, and clinically without neurological abnormalities, therefore, he was discharged from the hospital.

## DISCUSSION

This case report highlights the coexistence of TTP in a patient with an initial diagnosis of malaria caused by *P. vivax*, a tropical vector-borne disease that is a major public health problem in Colombia owing to its high prevalence. Malaria caused by *P. vivax* does not usually occur in severe forms, as is often the case with *P. falciparum*. Cases of TTP associated with severe malaria caused by *P. falciparum* in response to plasma exchange treatment have been described^7^.

Hematological alterations in malaria are well documented, with the prevalence of thrombocytopenia ranging from 60 to 80%^8^. The degree of coagulation disorder varies from mild to severe and correlates with parasitemia and the clinical severity of the disease, with 33-50% of patients presenting with disseminated intravascular coagulation^9^. Anemia is common in malarial infections and generally presents with normal erythrocyte indices and a low reticulocyte count. The pathogenesis of anemia is multifactorial and includes intravascular destruction of parasitized erythrocytes, extravascular removal, and suppression of the bone marrow with dyserythropoiesis. Infected red blood cells can obstruct the microvasculature, thereby causing organ damage. In certain cases, serum lactate dehydrogenase levels increase due to hemolysis and hepatic injury^10^ ^,^ ^11^.

These findings justify the initial consideration of the patient's hematological and clinical alterations in relation to complicated malaria. However, the lack of response to treatment, absence of high parasite load at diagnosis, and low prevalence of severe malaria caused by *P. vivax* led to a diagnosis of TTP, which was subsequently confirmed with substantially reduced levels of ADAMTS13. These values have not been documented in other secondary thrombotic microangiopathies, where the decrease is usually moderate.

Given that TTP is an extremely rare condition, it may not be suspected in the first instance, and its timely diagnosis and treatment may be delayed, leading to fatal outcomes in most patients. In this patient, there was a favorable response to the treatment, achieving normalization of organ function and receiving an immunosuppressive regimen to prevent relapse, with normal ADAMTS 13 levels at follow-up.

The coexistence of both diseases has not been described and it is unknown whether there is a causal relationship between them or if it constitutes an epiphenomenon given that Colombia has a high prevalence of malaria. The patient's initial symptoms were nonspecific; however, thrombocytopenia associated with microangiopathic hemolysis was suggestive of TTP. This case highlights the importance of considering TTP in patients with thrombocytopenia and microangiopathic hemolysis, even those with infections or conditions that trigger secondary thrombotic microangiopathy.
